# High-throughput sequencing technology reveals that continuous cropping of American ginseng results in changes in the microbial community in arable soil

**DOI:** 10.1186/s13020-017-0139-8

**Published:** 2017-07-03

**Authors:** Linlin Dong, Jiang Xu, Lianjuan Zhang, Juan Yang, Baosheng Liao, Xiwen Li, Shilin Chen

**Affiliations:** Institute of Chinese Materia Medica, China Academy of Chinese Medical Sciences, Beijing, 100700 China

**Keywords:** *Panax quinquefolius* L., Continuous cropping system, Bacterial community, Fungal community, High-throughput sequencing technology

## Abstract

**Background:**

American ginseng (*Panax quinquefolius* L.) is renowned worldwide for its eutherapeutic effects. The replantation of American ginseng usually fails due to problems associated with continuous cropping. An imbalance in the microbial community is thought to be responsible for this, but the overall changes in microbial communities under a continuous cropping system are unclear.

**Methods:**

This study used quantitative polymerase chain reaction combined with high-throughput sequencing methods to confirm changes in a microbial community under continuous cropping of American ginseng.

**Results:**

Copy numbers of bacteria and fungi significantly declined by 47.7 and 45.5%, respectively, upon American ginseng cropping over 3 years. A total of 66,391 classified sequences were obtained from high-throughput sequencing analyses of 16S and 18S rRNA in six soil samples. A decline in bacterial diversity and an increase in fungal diversity were observed in the continuous cropping soils of American ginseng compared to those of traditional crops. Compared with soils used for traditional crops, the relative abundance of bacterial and fungal groups changed in soils subjected to continuous cropping with American ginseng.

**Conclusions:**

Our results revealed that the diversity and composition of soil bacterial and fungal communities changed in the continuous cropping of American ginseng compared to those of traditional crops. Those data provided comprehensive insight into microbial communities at the agro-ecosystem scale and contributed to the understanding of micro-ecological environments in the rhizosphere of medicinal plants.

**Electronic supplementary material:**

The online version of this article (doi:10.1186/s13020-017-0139-8) contains supplementary material, which is available to authorized users.

## Background

American ginseng (*Panax quinquefolius* L.) is a remarkable medicine that is renowned globally for its eutherapeutic effects [1]. It is a perennial plant that is usually continuously cultivated for 3 years in China. However, the replantation of American ginseng usually fails due to problems associated with continuous cropping [2]. These problems include deterioration of the physicochemical properties of soil, autotoxicity, and changes in the soil microbial community [3, 4]. Among these, imbalances in soil microbial communities were shown to be the pivotal problem with continuous cropping system [5].

Soil microbial communities are abundant and play important roles in the biochemical cycles of terrestrial ecosystems [6]. The diversity and composition of soil microbial communities are critical for maintaining soil health and quality [7, 8]. Changes in the diversity and composition of microbial community are related to shifts in a series of biotic and/or abiotic factors such as the cropping system, plant species, and soil type [9, 10]. Many studies have reported that cropping systems have significant effects on the diversity and composition of microbial communities [5, 11]. For example, based on phospholipid fatty-acid analyses, American ginseng cultivation was shown to cause a decline in metabolism function and a shift in the structure of soil microbial communities in the rhizosphere [12]. Therefore, changes in the microbial community can affect soil micro-ecology and thus plant growth. High-throughput sequencing data revealed that fungal diversity declined and changes of microbial community were occurred in the soils of *Panax notoginseng* continuous cropping for 3 years [13]. However, few studies have simultaneously reported on the changes in bacterial and fungal communities that are associated with the continuous cropping of American ginseng.

In this study, our objective was to detect changes in the diversity and composition of bacterial and fungal communities under a continuous cropping system of American ginseng. The obtained results should be useful for understanding the effects of cropping systems on soil micro-ecology, contribute to the cultivation of medicinal plants, and improve the sustainable development of the medicinal industry.

## Methods

### The experiment and soil collection

This experiment was conducted in Huairou, Beijing (40°300′N, 116°600′E), which is one of the main areas of American ginseng production in China. American ginseng was cultivated on traditional farmlands, in which mainly maize had been planted for more than 30 years. In the process of maize cultivation, Organic fertilizer (3.0 kg m^−2^) was applied as base fertilizer. The soil moisture content was mediated according to the developmental stages of maize. American ginseng was cultivated following the standard operating procedures of Good Agricultural Practice [14, 15]. Organic fertilizer (2.5 kg m^−2^) was added in October each year. The soil moisture content is 40–50%. Field plots in our plantation were arranged in a fully randomized block design with three replicate plots (1.5 × 30 m). Six plants grown for 3 years were chosen from one plot of American ginseng farmland (AGF) and their soil samples in the rhizosphere were collected [16]. Soil samples from maize rhizospheres were used as controls (traditional farmland: TF). Six soil samples from one plot were combined to form a single sample. Those samples were AGF1, AGF2, AGF3, TF1, TF2 and TF3. In total, six samples were gathered, homogenized by being passed through a 2-mm sieve, and stored at −80 °C until subjected to further processing for the experiments. The characteristics of these soil samples are shown in Additional file 1: Table S1.

### DNA extraction and quantitative polymerase chain reaction (QPCR)

**Fig. 1:**
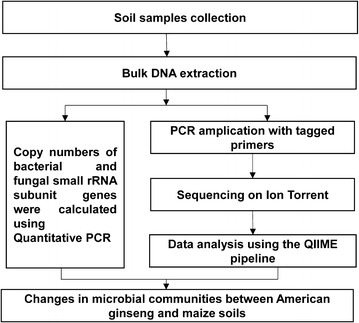
The flow chart of the experimental procedure

Total DNA was extracted from freeze-dried soil using the MoBio Powersoil Kit (MoBio, Inc., Carlsbad, CA, USA) based on the manufacturer’s instructions. Total DNA was used to analyze the changes in the microbial communities between American ginseng and maize soils. The flow chart of the experimental procedure was showed in Fig. 1. Copy numbers of bacterial and fungal small rRNA subunit genes were calculated as described by Fierer et al. [17] and Rousk et al. [18]. Briefly, fungal gene fragments were amplified using the 5.8S/ITS1f primer pair and bacterial gene fragments were amplified using the Eub338/Eub518 primer pair. Quantitative PCR was performed in a volume of 25 µL with the 2× SYBR Green PCR Master Mix (Takara Bio, Shiga, Japan) using an ABI7500 Fast Real-Time PCR system (Applied Biosystems, Foster City, CA, USA). Double-distilled water was used in the controls instead of template DNA. Copy numbers of bacterial and fungal genes were determined using a regression equation for each assay. Cycle threshold (*C*t) values were obtained from the known copy numbers in the standards.

### PCR amplification and Ion Torrent™ Sequencing

For each sample, the gene sequences of 16S rRNA and 18S rRNA were respectively amplified using conserved bacterial primers [17] and conserved fungal primers 817F/1196R [18]. The forward and reverse primers each contained a 10-bp barcode, as shown in Additional file 1: Table S2. PCR was carried out in quadruplicate in 25-µL reaction mixtures, each containing 1 µL (10 mM) of each forward and reverse primer, 2 µL of template DNA, 2.5 µL of reaction buffer, 2.5 µL of dNTP, and 0.2 µL of Taq DNA recombinant polymerase (Takara, Otsu, Japan). Samples were denatured at 94 °C for 3 min and then amplified using 28 cycles at 94 °C for 45 s, 50 °C for 30 s, and 72 °C for 60 s. A final extension of 10 min was added at the end of the program. Negative controls (no templates) were included to check for primer or sample DNA contamination. PCR products were excised from 1% agarose gel, purified using the MinElute Gel Extraction Kit (Qiagen, Valencia, CA, USA), and quantified using a Quant-iT PicoGreen dsDNA Assay Kit (Invitrogen, Carlsbad, CA, USA). The resulting amplicons were pooled in equimolar ratios. Sequencing was performed on an Ion Torrent™ Personal Genome Machine using the Ion Xpress Template Kit (Life Technologies, Carlsbad, CA, USA) and the Ion 314 chip (Life Technologies) following the manufacturer’s protocol.

### Data analysis

Data were processed as described by the QIIME pipeline [19]. In brief, bacterial and fungal sequences were trimmed and assigned to different samples based on their barcodes. Sequences were binned into operational taxonomic units (OTUs) at the 97% similarity level. Representative sequence alignments were generated through assignment with PyNAST, sequence alignment was performed to remove gaps, and locations known to be excessively variable were filtered [20]. The filtered alignment sequences were then used to build a phylogenetic tree using FastTree [21]. The taxonomic identities of bacteria and fungi were respectively determined using the RDP [22] and Sliva schemes [23]. Alpha diversities of bacterial and fungal communities were determined to assay phylogenetic diversity, along with Shannon indices (*H*′), Chao 1, and observed species by a modified version of the procedure described by Caporaso et al. [19]. Pairwise distances between communities were determined using Weighted Unifrac values, and the results of microbial communities were visualized in a principal coordinate analysis (PCoA) matrix.

### Statistical analyses

SPSS version 16.0 was used for statistical analyses of the diversity and relative abundance of soil microbial communities (SPSS Inc., Chicago, IL, USA). The parameters were calculated for all replicates and subjected to an analysis of variance by one-way ANOVA. The value of each bar represents the mean ± SD of *n* = 3. Mean values were reported as significant or non-significant by paired *t*-tests (*P* < 0.05).

The Minimum Standards of Reporting Checklist contains details of the experimental design, and statistics, and resources used in this study (Additional file 2).

## Results

### Estimation of the ratio of fungi to bacteria by quantitative PCR

**Fig. 2:**
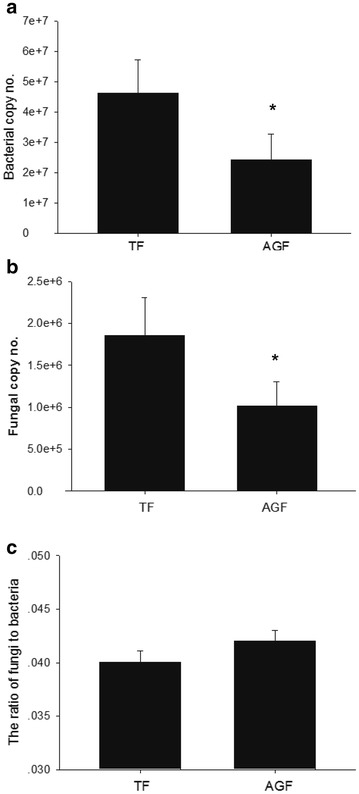
Copy numbers of bacteria (**a**), fungi (**b**) and the ratio of fungi to bacteria (**c**). TF and AGF refer to soil samples from traditional farmland and American ginseng farmland, respectively. The value of each bar represents the mean ± SD of *n* = 3. An asterisk denotes a significant difference between TF and AGF at *P* < 0.05

The copy numbers of bacterial and fungal communities had significantly declined by 47.7 and 45.5%, respectively, in soils subjected to continuous cropping with American ginseng compared with those of traditional cropping system (Fig. 2a, b). Additionally, the ratio of fungi to bacteria showed an increasing trend in soils of American ginseng compared to those of traditional crops (maize) (Fig. 2c).

### Bacterial diversity was lower in soils used for cultivating American ginseng

**Fig. 3:**
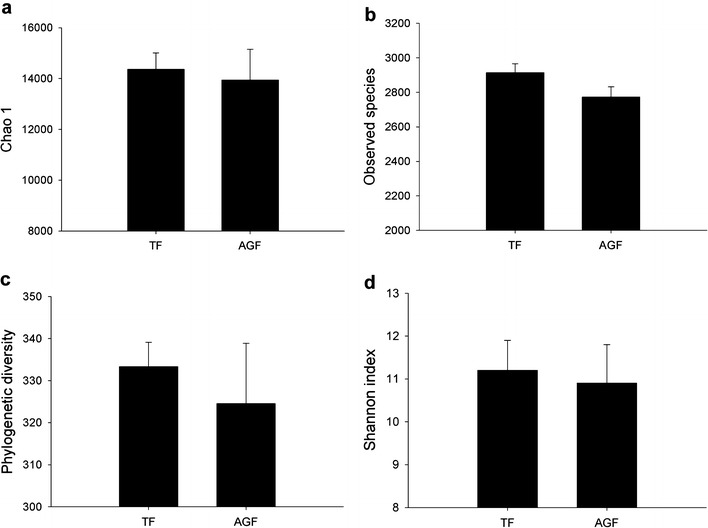
Changes of Chao 1 (**a**), observed species (**b**), phylogenetic diversity (**c**), and Shannon index (**d**) for the 16S rRNA gene sequences from soils used for traditional crops and American ginseng. TF and AGF refer to soil samples from traditional farmland and American ginseng farmland, respectively. The value of each *bar* represents the mean ± SD of *n* = 3

A total of 45,788 sequences were obtained by high-throughput sequencing analyses of 16S rRNA gene sequences, with an average of 7631 sequences per sample (range 5812–9458; most common length range 220–229 bp) (Additional file 1: Table S3). Chao 1, observed species, phylogenetic diversity, and Shannon index showed decreasing trends in soils used for American ginseng cultivation compared with those in the traditional cropping system (Fig. 3).

### Fungal diversity was higher in soils used for cultivating American ginseng

**Fig. 4:**
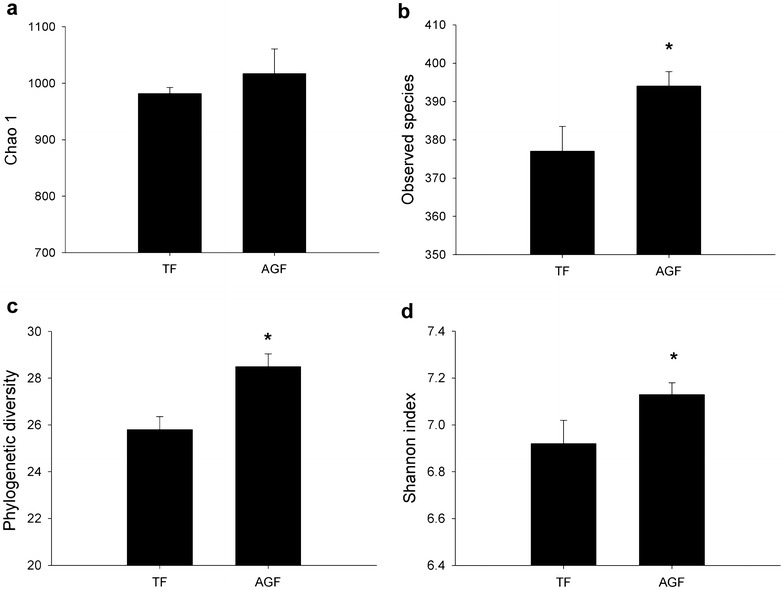
Changes of Chao 1 (**a**), observed species (**b**), phylogenetic diversity (**c**), and Shannon index (**d**) for the 18S rRNA gene sequences from soils used for traditional crops and American ginseng. TF and AGF refer to soil samples from traditional farmland and American ginseng farmland, respectively. An *asterisk* denotes a significant difference between TF and AGF at *P* < 0.05. The value of each *bar* represents the mean ± SD of *n* = 3

A total of 20,603 sequences were obtained in high-throughput sequencing analyses of 18S rRNA gene sequences (Additional file 1: Table S3). The average number of sequences per sample was 3433 (range 1454–5563; most common length range 225–282 bp). Chao 1, observed species, phylogenetic diversity, and Shannon index showed increasing trends in soils used to cultivate American ginseng compared with those of traditional cropping system (Fig. 4).

### Changes of bacterial composition in soils used to cultivate American ginseng

**Fig. 5:**
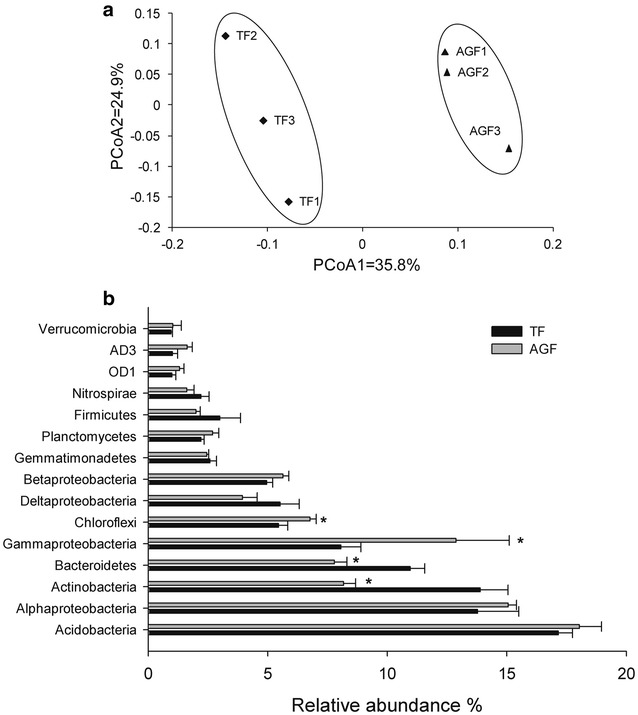
Changes in the composition of the bacterial community in soils used for traditional crops and American ginseng. **a** PCoA ordination plots show the relatedness of samples that were separated using weighted UniFrac distances of classified 16S rRNA gene sequences. **b** The relative abundances of the bacterial community (>1%) at the phylum level. TF and AGF refer to soil samples from traditional farmland and American ginseng farmland, respectively. An asterisk denotes a significant difference between TF and AGF at *P* < 0.05. The value of each *bar* represents the mean ± SD of *n* = 3

Bacterial composition differed significantly between soils used for American ginseng cultivation and those of maize cultivation (Fig. 5). PCoA ordination showed variations in the bacterial community among soils used for cultivating American ginseng and for maize (Fig. 5a). The first principal component (35.8% contribution) and the second principal component axes (24.9% contribution) differentiated the bacterial communities in soils used for cultivating American ginseng and maize.

The distribution of bacterial groups at the phylum level differed between the American ginseng and maize soils (Fig. 5b). Specifically, the relative abundances of Acidobacteria, Alphaproteobacteria, Gammaproteobacteria, Chloroflexi, Betaproteobacteria, Planctomycetes, OD1, AD3, and Verrucomicrobia were higher in soils used to cultivate American ginseng than in those used for maize cultivation. Meanwhile, the relative abundances of Actinobacteria, Bacteroidetes, Deltaproteobacteria, Gemmatimonadetes, Firmicutes, and Nitrospirae were lower in the American ginseng soils.

### Changes in the fungal composition of soils used for American ginseng cultivation

**Fig. 6:**
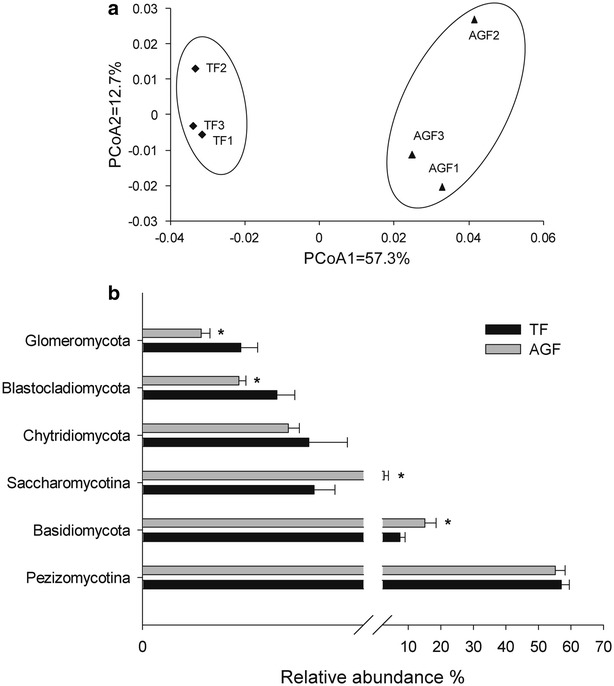
Changes in the composition of the fungal community in soils of traditional farmland and American ginseng farmland. **a** PCoA ordination plots show the relatedness of samples that were separated using weighted UniFrac distances of classified 18S rRNA gene sequences. **b** The relative abundances of the fungal community at the phylum level. TF and AGF refer to soil samples from traditional farmland and American ginseng farmland, respectively. An *asterisk* denotes a significant difference between TF and AGF at *P* < 0.05. The value of each *bar* represents the mean ± SD of *n* = 3

Fungal compositions also differed between American ginseng and maize soils (Fig. 6). These compositions showed variations based on PCoA ordination analyses (Fig. 6a). The first principal component (57.3% contribution) and second principal component axes (12.7% contribution) differentiated the fungal composition of American ginseng and maize soils.

In addition, the distribution of fungal communities varied between soils used for cultivating American ginseng and those of maize at the phylum level (Fig. 6b). Compared with soils used for maize cultivation, the relative abundances of Pezizomycotina, Chytridiomycota, Blastocladiomycota, and Glomeromycota were lower and the relative abundances of Basidiomycota and Saccharomycotina were higher in the American ginseng soils.

### The relative abundance of bacterial groups changed in soils of American ginseng cultivation

**Fig. 7:**
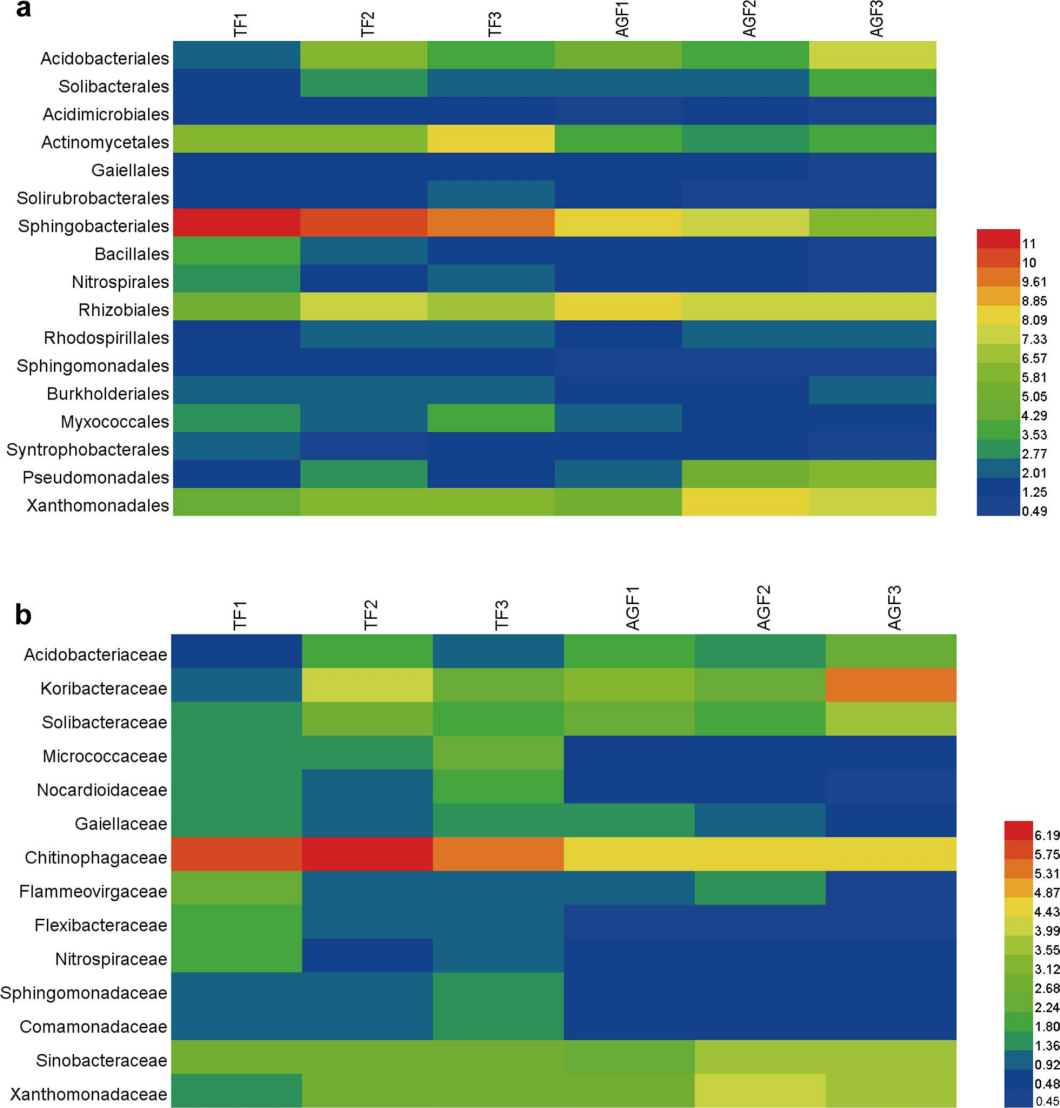
The relative abundance of main bacterial groups from soils used for American ginseng and traditional crops at the order level (**a**) and family level (**b**). The relative abundance (>0.5%) of bacterial groups of each sample at the order lever, and the relative abundance (>0.4%) of each sample at the family level. TF and AGF refer to soil samples from traditional farmland and American ginseng farmland, respectively

Bacterial groups also differed between the American ginseng and maize soils at the levels of order and family (Fig. 7; Additional file 1: Tables S4, S5). The relative abundances of Acidimicrobiales, Actinomycetales, Gaiellales, Solirubrobacterales, Sphingobacteriales, Bacillales, Nitrospirales, Sphingomonadales, Burkholderiales, Myxococcales, and Syntrophobacterales declined by 0.1–49.6% in soils used to cultivate American ginseng compared with those of maize cultivation (Fig. 7a). However, the relative abundances of Acidobacteriales, Solibacterales, Rhizobiales, Rhodospirillales, Pseudomonadales, and Xanthomonadales increased by 8.1–160%. In soils used for American ginseng cultivation, the relative abundances of Micrococcaceae, Nocardioidaceae, Gaiellaceae, Chitinophagaceae, Flammeovirgaceae, Flexibacteraceae, Nitrospiraceae, Sphingomonadaceae, and Comamonadaceae decreased by 21.5–85.7% compared to those of maize cultivation. Conversely, the relative abundances of Acidobacteriaceae, Koribacteraceae, Solibacteraceae, Sinobacteraceae, and Xanthomonadaceae increased by 21.0–86.0% (Fig. 7b).

### The relative abundance of fungal groups changes in soils cultivated with American ginseng

**Fig. 8:**
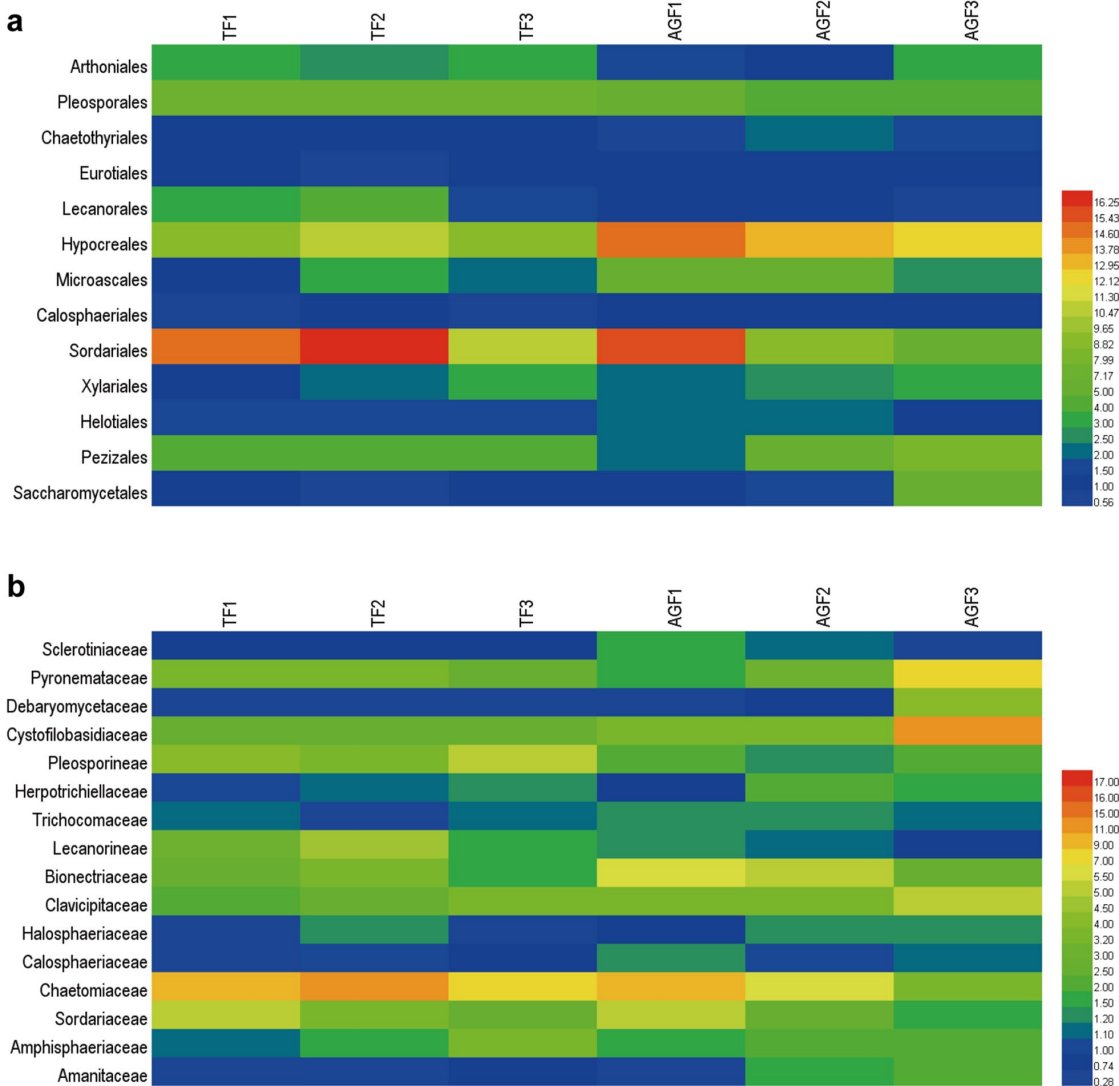
The relative abundance of main fungal taxa from soils used for American ginseng and traditional crops at the order level (**a**) and family level (**b**). The relative abundance (>0.5%) of fungal taxa of each sample at the order level, and the relative abundance (>0.2%) of each sample at the family level. TF and AGF refer to soil samples from traditional farmland and American ginseng farmland, respectively

Fungal taxa differed between the soils of American ginseng and maize at the levels of order and family (Fig. 8; Additional file 1: Table S6). Compared with soils used for maize cultivation, the relative abundances of Arthoniales, Pleosporales, Lecanorales, and Sordariales declined by 61.4–274% in soils of American ginseng cultivation. In contrast, the relative abundances of Chaetothyriales, Eurotiales, Hypocreales, Microascales, Calosphaeriales, Xylariales, Helotiales, Pezizales, and Saccharomycetales increased by 13.4–132% in soils used for American ginseng cultivation (Fig. 8a). Furthermore, the relative abundances of Sclerotiniaceae, Pyronemataceae, Debaryomycetaceae, Cystofilobasidiaceae, Herpotrichiellaceae, Trichocomaceae, Bionectriaceae, Clavicipitaceae, Halosphaeriaceae, Calosphaeriaceae, and Amanitaceae increased by 28.1–215%, and the relative abundances of Pleosporineae, Lecanorineae, Chaetomiaceae, Sordariaceae, and Amphisphaeriaceae showed declining trends (0.6–58.5%) in soils of American ginseng cultivation compared with those of maize cultivation (Fig. 8b).

## Discussion

The increase in the ratio of fungi to bacteria was observed in the soils of American ginseng cultivation compared to those of maize in our study. Many studies have reported that the ratios of fungi to bacteria were altered in cropping systems [24, 25]. The ratio of fungi to bacteria significantly increased in the continuous cropping of *P. notoginseng* [13]. These conclusions are also supported by our results. The ratio of fungi to bacteria is the most important characteristic of soil functionality and can serve as an indicator of ecosystem processes [26]. These results could imply soil functionality was changed in a continuous cropping system.

Our results confirmed a decline in bacterial diversity and an increase in fungal diversity in soils subjected to a continuous cropping system of American ginseng. As mentioned above, phylotype diversity of fungi also increased during continuous cropping of peanut [5]. Benizri et al. [27] reported that the bacterial diversity changed in the continuous cropping soil of peaches. It has been proposed that the diversity of soil microorganisms is related to the maintenance of soil health and quality [8]. Fungal diversity was associated with the death rate of *P. notoginseng* in the continuous cropping system [13]. Fungal diversity has been suggested to have beneficial effects on plant production by suppressing pathogens [28]. A decrease in soil microbial diversity was responsible for the development of soil-borne plant diseases [29]. Taking the obtained findings together, the changes of microbial diversity in continuous cropping of American ginseng could alter the micro-ecological environment.

Cropping systems changed the composition of soil microorganisms and affected the soil health and quality [11, 30, 31]. Significant changes of microbial communities were observed in rhizosphere soils during the continuous cultivation of ginseng [32]. Fungal populations exhibited significant dynamic changes in the continuous cropping of peanut, and both pathogenic and beneficial fungi were positively selected over time [5]. Plant species is thought to select specific microbial populations in the rhizosphere and root exudates is a driving force [8, 33, 34]. American ginseng is a perennial plant and its root exudates accumulate in the rhizosphere, possibly presenting substrates for several groups of bacterial communities. For instance, Proteobacteria and Bacteroidetes increased in relative abundance in high-nitrogen plots [6], which may explain the increase in the proportions of their abundances. However, plants do not only provide nutrients for microbial communities, but also contain a series of antimicrobial metabolites in their root exudates [10, 33, 34]. This may explain observed decreases in the relative abundances of microbial communities. Additionally, changes in soil chemical properties are likely to drive changes in microbial community composition [24]. Our analyses did not show significant differences in pH, total nitrogen, available potassium, or organic matter between the soils of traditional cropping and American ginseng continuous cropping (Additional file 1: Table S1). Additionally, the contents of Olsen-phosphatase were significant difference in soils of traditional cropping compared to those of American ginseng, which effects on the microbial communities would need to further verification. Thus, cropping system was speculated as a dominant factor in disrupting the balance of soil microbial communities [13]. Changes in the composition of bacterial community may lead to variations in metabolic capacity, biodegradation, and disease-suppression abilities [8, 35]. The negative impact of continuous cropping on soil productivity has been shown [36]. These findings indicate that continuous cropping might have negative effects on ecosystem processes that are central to key ecosystem services. In this study, high-throughput sequencing analyses provided simultaneous and detailed insight into the bacterial and fungal communities in the agro-ecosystem of medicinal plants. HerbGenomics has been provided as a new discipline and metagenomic analyses of soil microbial populations is an important part of this discipline [37].

## Conclusions

In summary, compared to those of traditional cropping system, the bacterial diversity decreased and the fungal diversity showed increasing trends in soils used for the continuous cropping of American ginseng; meanwhile, the composition of bacterial and fungal communities changed in soils cultivated with American ginseng. Our work will be of great significance for the understanding of continuous cropping obstacles from rhizospheric microorganisms, promoting the continuous development of medicinal plants and accelerating the recycling of soil resources.

## Additional files

**Additional file 1: Table S1–S6.** The supplementary information for this article.

**Additional file 2.** Minimum Standards of Reporting Checklist.

## Abbreviations

TF: traditional farmland
AGF: American ginseng farmland
rRNA: ribosomal RNA
PCR: polymerase chain reaction
QPCR: quantitative polymerase chain reaction
PCoA: principal coordinates analysis
RDP: ribosomal database project
OTUs: operational taxonomic units
