# Supplementary material for: High-throughput sequencing technology reveals that continuous cropping of American ginseng results in changes in the microbial community in arable soil
Source: Chin Med. 2017 Jul 3;12:18. doi: 10.1186/s13020-017-0139-8 (PMC5496220; doi:10.1186/s13020-017-0139-8)
Supplement: Supplementary file 1 — Additional file 1: Table S1–S6. The supplementary information for this article. [file 13020_2017_139_MOESM1_ESM.doc]

**Table S1** Soil chemical characteristics between soil samples of traditional farmland and American ginseng farmland

| Soils | pH | Total N  (g kg^-1^) | Olsen-P  (g kg^-1^) | Available K  (mg kg^-1^) | Organic matter  (g kg^-1^) |
| --- | --- | --- | --- | --- | --- |
| TF | 6.89±0.06 | 0.94±0.05 | 25.3±1.42 | 119±11.3 | 16.4±0.94 |
| AGF | 6.64±0.02 | 0.96±0.02 | 10.47±1.05* | 126±6.05 | 16.7±0.43 |

TF: Traditional farmland; AGF: American ginseng farmland. Each data was presented the mean ± SD of *n* = 3, and the asterisk stand for significant in the treatments with the control at 0.05 level.

**Table S2** List of the 10-bp barcodes used to tag each PCR of bacterial production analyzed

| acgttaccgt | agtcgagaga |
| --- | --- |
| actcacagag | agtctgactg |
| agcactgtag | agtgacacac |

**Table S3** Bacterial and fungal numbers of sequences, derived OTUs and average length in each sample

| Samples | Bacterial community | | | |  | Fungal community | | |
| --- | --- | --- | --- | --- | --- | --- | --- | --- |
|  | Sequences | OTUs | Average Length | |  | Sequences | OTUs | Average Length |
| TF1 | 8096 | 5836 | | 229 |  | 2972 | 813 | 280 |
| TF2 | 9458 | 6481 | | 222 |  | 3396 | 834 | 282 |
| TF3 | 5812 | 4500 | | 224 |  | 2922 | 801 | 281 |
| AGF1 | 6749 | 4784 | | 228 |  | 4296 | 1060 | 274 |
| AGF2 | 8386 | 5904 | | 223 |  | 5563 | 1266 | 260 |
| AGF3 | 7293 | 4919 | | 220 |  | 1454 | 499610000000000000000000000000000000000000000000000000000000000000000000000000000000000000000000000000000000000000000000000000000 | 225 |

TF: Traditional farmland; AGF: American ginseng farmland.

**Table S4** The relative abundance (<0.5%) of bacterial groups of each sample at the order lever.

| Taxon | TF | AGF |
| --- | --- | --- |
| Micrococcales | 0.084±0.027 | 0.070±0.023 |
| Euzebyales | 0.033±0.009 | 0.009±0.005* |
| Armatimonadales | 0.062±0.016 | 0.033±0.017 |
| Chthonomonadales | 0.028±0.007 | 0.170±0.083* |
| Bacteroidales | 0.047±0.020 | 0.055±0.026 |
| Flavobacteriales | 0.098±0.033 | 0.103±0.032 |
| Chlamydiales | 0.018±0.011 | 0.005±0.005 |
| Chlorobiales | 0.000±0.000 | 0.004±0.004 |
| Anaerolineales | 0.009±0.005 | 0.013±0.007 |
| Caldilineales | 0.258±0.077 | 0.148±0.050* |
| Chloroflexales | 0.069±0.030 | 0.014±0.009* |
| Roseiflexales | 0.449±0.031 | 0.536±0.146 |
| Dehalococcoidales | 0.004±0.004 | 0.014±0.008 |
| Ktedonobacterales | 0.025±0.019 | 0.018±0.005 |
| Thermogemmatisporales | 0.297±0.074 | 0.991±0.593* |
| Thermobaculales | 0.065±0.024 | 0.112±0.034 |
| Thermomicrobiales | 0.008±0.008 | 0.004±0.004 |
| Chlorophyta | 0.065±0.036 | 0.009±0.009* |
| Stramenopiles | 0.062±0.011 | 0.059±0.026 |
| Streptophyta | 0.353±0.051 | 0.698±0.460* |
| Nostocales | 0.061±0.021 | 0.004±0.004 |
| Chroococcales | 0.105±0.011 | 0.004±0.004* |
| Oscillatoriales | 0.135±0.042 | 0.005±0.005* |
| Pseudanabaenales | 0.272±0.021 | 0.014±0.014* |
| Elusimicrobiales | 0.096±0.036 | 0.209±0.043* |
| Fibrobacterales | 0.004±0.004 | 0.005±0.005 |
| Haloplasmatales | 0.000±0.000 | 0.004±0.004 |
| Lactobacillales | 0.015±0.008 | 0.096±0.096* |
| Turicibacterales | 0.000±0.000 | 0.005±0.005 |
| Clostridiales | 0.208±0.023 | 0.237±0.009 |
| Coriobacteriales | 0.008±0.004 | 0.008±0.008 |
| Halanaerobiales | 0.006±0.006 | 0.005±0.005 |
| Natranaerobiales | 0.004±0.004 | 0.009±0.005 |
| Fusobacteriales | 0.000±0.000 | 0.004±0.004 |
| Gemmatimonadales | 0.038±0.002 | 0.107±0.079* |
| Phycisphaerales | 0.175±0.017 | 0.233±0.090 |
| Gemmatales | 0.698±0.081 | 1.082±0.226* |
| Pirellulales | 0.015±0.010 | 0.087±0.053 |
| Planctomycetales | 0.000±0.000 | 0.037±0.037 |
| Caulobacterales | 0.271±0.051 | 0.305±0.060 |
| Rhodobacterales | 0.585±0.039 | 0.234±0.049* |
| Rickettsiales | 0.096±0.016 | 0.131±0.046 |
| Gallionellales | 0.293±0.039 | 0.730±0.228* |
| Hydrogenophilales | 0.011±0.011 | 0.009±0.004 |
| Methylophilales | 0.064±0.020 | 0.121±0.032* |
| Neisseriales | 0.004±0.004 | 0.005±0.005 |
| Nitrosomonadales | 0.168±0.040 | 0.136±0.024 |
| Procabacteriales | 0.000±0.000 | 0.009±0.004 |
| Rhodocyclales | 0.045±0.012 | 0.051±0.014 |
| Bdellovibrionales | 0.171±0.029 | 0.077±0.028* |
| Desulfobacterales | 0.009±0.005 | 0.005±0.005 |
| Desulfovibrionales | 0.004±0.004 | 0.005±0.005 |
| Desulfuromonadales | 0.037±0.013 | 0.008±0.008* |
| Spirobacillales | 0.150±0.043 | 0.109±0.048 |
| Acidithiobacillales | 0.008±0.008 | 0.010±0.005 |
| Alteromonadales | 0.048±0.020 | 0.037±0.019 |
| Chromatiales | 0.069±0.003 | 0.062±0.017 |
| Enterobacteriales | 0.044±0.015 | 0.054±0.035 |
| Legionellales | 0.338±0.052 | 0.584±0.051* |
| Methylococcales | 0.011±0.011 | 0.031±0.008 |
| Oceanospirillales | 0.019±0.008 | 0.028±0.009 |
| Thiotrichales | 0.007±0.007 | 0.005±0.005 |
| Vibrionales | 0.004±0.004 | 0.009±0.005 |
| Spirochaetales | 0.000±0.000 | 0.013±0.008 |
| Synergistales | 0.016±0.011 | 0.008±0.008 |
| Anaeroplasmatales | 0.480±0.110 | 0.414±0.068 |
| Opitutales | 0.075±0.007 | 0.063±0.030 |
| Verrucomicrobiales | 0.069±0.022 | 0.030±0.010 |

TF and AGF refer to soil samples from traditional farmland and American ginseng farmland, respectively. An asterisk denotes a significant difference between TF and AGF at *P* < 0.05. The value of each bar represents the mean ± SD of *n*=3.

**Table S5** The relative abundance (<0.4%) of bacterial groups of each sample at the family level

| Taxon | TF | AGF |
| --- | --- | --- |
| Iamiaceae | 0.051±0.008 | 0.004±0.004* |
| Microthrixaceae | 0.008±0.004 | 0.005±0.005 |
| Actinosynnemataceae | 0.106±0.018 | 0.030±0.010* |
| Cellulomonadaceae | 0.092±0.009 | 0.045±0.009 |
| Corynebacteriaceae | 0.004±0.004 | 0.022±0.004* |
| Frankiaceae | 0.022±0.012 | 0.043±0.022 |
| Geodermatophilaceae | 0.179±0.016 | 0.053±0.006* |
| Intrasporangiaceae | 0.256±0.046 | 0.173±0.053 |
| Microbacteriaceae | 0.592±0.074 | 0.251±0.043* |
| Micrococcaceae | 1.887±0.257 | 0.635±0.058* |
| Micromonosporaceae | 0.277±0.055 | 0.271±0.081 |
| Mycobacteriaceae | 0.107±0.023 | 0.157±0.014 |
| Nocardiaceae | 0.083±0.012 | 0.052±0.010 |
| Nocardiopsaceae | 0.015±0.009 | 0.005±0.005 |
| Promicromonosporaceae | 0.044±0.004 | 0.023±0.009 |
| Propionibacteriaceae | 0.368±0.074 | 0.170±0.061* |
| Pseudonocardiaceae | 0.168±0.047 | 0.126±0.040 |
| Sporichthyaceae | 0.089±0.009 | 0.099±0.008 |
| Streptomycetaceae | 0.488±0.049 | 0.317±0.049 |
| Streptosporangiaceae | 0.110±0.004 | 0.079±0.011 |
| Thermomonosporaceae | 0.077±0.011 | 0.021±0.011 |
| Euzebyaceae | 0.033±0.009 | 0.009±0.005 |
| Rubrobacteraceae | 0.811±0.108 | 0.230±0.118* |
| Conexibacteraceae | 0.067±0.007 | 0.197±0.129 |
| Patulibacteraceae | 0.106±0.005 | 0.027±0.002* |
| Solirubrobacteraceae | 0.457±0.141 | 0.147±0.018 |
| Armatimonadaceae | 0.053±0.019 | 0.010±0.005 |
| Chthonomonadaceae | 0.028±0.007 | 0.170±0.083* |
| Cryomorphaceae | 0.012±0.012 | 0.005±0.005 |
| Flavobacteriaceae | 0.085±0.031 | 0.098±0.037 |
| Amoebophilaceae | 0.006±0.006 | 0.023±0.006 |
| Balneolaceae | 0.025±0.006 | 0.025±0.018 |
| Ekhidnaceae | 0.004±0.004 | 0.000±0.000 |
| Saprospiraceae | 0.173±0.026 | 0.226±0.108 |
| Sphingobacteriaceae | 0.759±0.068 | 0.878±0.165 |
| Waddliaceae | 0.004±0.004 | 0.000±0.000 |
| Caldilineaceae | 0.258±0.077 | 0.148±0.050* |
| Chloroflexaceae | 0.020±0.010 | 0.005±0.005 |
| Oscillochloridaceae | 0.018±0.011 | 0.000±0.000 |
| Kouleothrixaceae | 0.275±0.055 | 0.393±0.100 |
| Roseiflexaceae | 0.124±0.038 | 0.130±0.043 |
| Thermogemmatisporaceae | 0.281±0.082 | 0.909±0.551 |
| Thermobaculaceae | 0.065±0.024 | 0.112±0.034 |
| Cyanobacteriaceae | 0.023±0.006 | 0.004±0.004 |
| Phormidiaceae | 0.135±0.042 | 0.005±0.005* |
| Pseudanabaenaceae | 0.272±0.021 | 0.014±0.014 |
| Fibrobacteraceae | 0.004±0.004 | 0.005±0.005 |
| Alicyclobacillaceae | 0.096±0.049 | 0.023±0.013 |
| Paenibacillaceae | 0.442±0.081 | 0.250±0.065 |
| Planococcaceae | 0.277±0.046 | 0.091±0.021* |
| Staphylococcaceae | 0.004±0.004 | 0.019±0.012 |
| Thermoactinomycetaceae | 0.066±0.023 | 0.035±0.010 |
| Streptococcaceae | 0.004±0.004 | 0.009±0.012 |
| Clostridiaceae | 0.101±0.008 | 0.107±0.020 |
| Lachnospiraceae | 0.038±0.015 | 0.022±0.017 |
| Peptococcaceae | 0.016±0.016 | 0.013±0.005 |
| Peptostreptococcaceae | 0.013±0.002 | 0.022±0.005* |
| Ruminococcaceae | 0.004±0.004 | 0.013±0.005 |
| Sulfobacillaceae | 0.009±0.005 | 0.013±0.007 |
| Symbiobacteriaceae | 0.012±0.007 | 0.005±0.005 |
| Syntrophomonadaceae | 0.006±0.006 | 0.005±0.005 |
| Veillonellaceae | 0.004±0.004 | 0.010±0.005 |
| Halanaerobiaceae | 0.006±0.006 | 0.005±0.005 |
| Phycisphaeraceae | 0.004±0.004 | 0.013±0.007 |
| Gemmataceae | 0.597±0.055 | 0.849±0.192 |
| Isosphaeraceae | 0.101±0.029 | 0.233±0.035* |
| Pirellulaceae | 0.015±0.010 | 0.083±0.055 |
| Caulobacteraceae | 0.259±0.058 | 0.287±0.055 |
| Beijerinckiaceae | 0.113±0.020 | 0.097±0.005 |
| Brucellaceae | 0.019±0.011 | 0.026±0.007 |
| Methylobacteriaceae | 0.011±0.011 | 0.026±0.016 |
| Methylocystaceae | 0.130±0.027 | 0.147±0.023 |
| Phyllobacteriaceae | 0.075±0.032 | 0.112±0.034 |
| Rhizobiaceae | 0.091±0.026 | 0.095±0.033 |
| Rhodobiaceae | 0.184±0.038 | 0.221±0.075 |
| Xanthobacteraceae | 0.110±0.033 | 0.032±0.025 |
| Hyphomonadaceae | 0.100±0.035 | 0.155±0.030 |
| Rhodobacteraceae | 0.485±0.052 | 0.079±0.020* |
| Acetobacteraceae | 0.199±0.032 | 0.333±0.093* |
| Pelagibacteraceae | 0.008±0.004 | 0.005±0.005 |
| Rickettsiaceae | 0.010±0.005 | 0.005±0.005 |
| Erythrobacteraceae | 0.087±0.014 | 0.027±0.009* |
| Alcaligenaceae | 0.374±0.058 | 0.400±0.043* |
| Burkholderiaceae | 0.165±0.034 | 0.353±0.194* |
| Oxalobacteraceae | 0.562±0.114 | 0.351±0.026* |
| Methylophilaceae | 0.064±0.016 | 0.121±0.024* |
| Nitrosomonadaceae | 0.168±0.040 | 0.136±0.024 |
| Procabacteriaceae | 0.000±0.000 | 0.009±0.004 |
| Rhodocyclaceae | 0.045±0.012 | 0.051±0.014 |
| Bacteriovoracaceae | 0.040±0.008 | 0.014±0.009 |
| Bdellovibrionaceae | 0.127±0.034 | 0.054±0.033 |
| Nitrospinaceae | 0.009±0.005 | 0.005±0.005 |
| Desulfovibrionaceae | 0.004±0.004 | 0.005±0.005 |
| Cystobacteraceae | 0.119±0.033 | 0.051±0.027* |
| Cystobacterineae | 0.012±0.007 | 0.010±0.005 |
| Haliangiaceae | 0.370±0.039 | 0.325±0.039 |
| Myxococcaceae | 0.064±0.016 | 0.053±0.024 |
| Nannocystaceae | 0.023±0.012 | 0.066±0.046 |
| Polyangiaceae | 0.209±0.053 | 0.120±0.031* |
| Desulfobacteraceae | 0.012±0.007 | 0.016±0.016 |
| Syntrophaceae | 0.060±0.020 | 0.062±0.011 |
| Syntrophorhabdaceae | 0.004±0.004 | 0.000±0.000 |
| Helicobacteraceae | 0.000±0.000 | 0.004±0.004 |
| Alteromonadaceae | 0.028±0.012 | 0.018±0.009 |
| Chromatiaceae | 0.010±0.005 | 0.004±0.004 |
| Ectothiorhodospiraceae | 0.023±0.006 | 0.028±0.016 |
| Enterobacteriaceae | 0.044±0.015 | 0.054±0.035 |
| Coxiellaceae | 0.259±0.049 | 0.399±0.068* |
| Legionellaceae | 0.020±0.006 | 0.065±0.011 |
| Methylococcaceae | 0.011±0.011 | 0.026±0.006 |
| Moraxellaceae | 1.683±0.404 | 4.124±1.198* |
| Pseudomonadaceae | 0.235±0.033 | 0.520±0.109* |
| Vibrionaceae | 0.004±0.004 | 0.009±0.005 |
| Spirochaetaceae | 0.000±0.000 | 0.013±0.008 |
| Leptospiraceae | 0.000±0.000 | 0.010±0.005 |
| Anaeroplasmataceae | 0.480±0.110 | 0.414±0.068 |
| Deinococcaceae | 0.013±0.007 | 0.000±0.000 |
| Verrucomicrobiaceae | 0.069±0.022 | 0.030±0.010 |

TF and AGF refer to soil samples from traditional farmland and American ginseng farmland, respectively. An asterisk denotes a significant difference between TF and AGF at *P* < 0.05. The value of each bar represents the mean ± SD of *n*=3.

**Table S6** The relative abundance (<0.2%) of fungal groups of each sample

| Taxon | TF | AGF |
| --- | --- | --- |
| Blastocladiaceae | 0.165±0.026 | 0.094±0.047 |
| Physodermataceae | 0.063±0.047 | 0.023±0.023 |
| Chytridiaceae | 0.018±0.009 | 0.026±0.016 |
| Megachytriaceae | 0.000±0.000 | 0.020±0.011* |
| Rhizophydiaceae | 0.022±0.011 | 0.023±0.023 |
| Terramycetaceae | 0.000±0.000 | 0.012±0.012 |
| Spizellomycetaceae | 0.352±0.062 | 0.148±0.008* |
| Monoblepharidaceae | 0.000±0.000 | 0.006±0.006 |
| Laboulbeniomycetes | 0.101±0.009 | 0.128±0.071 |
| Lichinomycetes | 0.063±0.002 | 0.058±0.011 |
| Orbiliomycetes | 0.082±0.018 | 0.049±0.013 |
| Neolectales | 0.014±0.010 | 0.000±0.000 |
| Pneumocystidomycetes | 0.000±0.000 | 0.006±0.006 |
| Schizosaccharomycetes | 0.047±0.029 | 0.026±0.016 |
| Taphrinomycetes | 0.011±0.011 | 0.000±0.000 |
| Anguillospora | 0.082±0.018 | 0.051±0.031 |
| Lecophagus | 0.010±0.010 | 0.066±0.017* |
| Neoplaconema | 0.036±0.032 | 0.012±0.012 |
| Phaeomoniella | 0.018±0.009 | 0.012±0.012 |
| Phoma | 0.059±0.016 | 0.052±0.017 |
| Pulchromyces | 0.084±0.012 | 0.000±0.000* |
| Sirococcus | 0.062±0.021 | 0.070±0.001 |
| Dacrymycetes | 0.000±0.000 | 0.006±0.006 |
| Wallemiomycetes | 0.000±0.000 | 0.041±0.021* |
| Agaricostilbomycetes | 0.025±0.021 | 0.014±0.007 |
| Cystobasidiomycetes | 0.074±0.024 | 0.028±0.014* |
| Microbotryomycetes | 0.003±0.003 | 0.138±0.035* |
| Pucciniomycetes | 0.029±0.003 | 0.008±0.008 |
| Exobasidiomycetes | 0.041±0.009 | 0.072±0.011 |
| Ustilaginomycetes | 0.075±0.029 | 0.018±0.018* |
| Entomophthorales | 0.019±0.010 | 0.029±0.021 |
| Harpellales | 0.000±0.000 | 0.012±0.012 |
| Kickxellales | 0.072±0.033 | 0.194±0.064* |
| Endogonales | 0.012±0.009 | 0.049±0.027 |
| Mortierellales | 0.039±0.008 | 0.083±0.042 |
| Zoopagales | 0.177±0.021 | 0.169±0.020 |
| Ambisporaceae | 0.057±0.039 | 0.020±0.011 |
| Acaulosporaceae | 0.215±0.129 | 0.101±0.031* |
| Pacisporaceae | 0.000±0.000 | 0.014±0.007 |
| Scutellosporaceae | 0.000±0.000 | 0.008±0.008 |
| Paraglomeraceae | 0.060±0.038 | 0.012±0.012 |

TF and AGF refer to soil samples from traditional farmland and American ginseng farmland, respectively. An asterisk denotes a significant difference between TF and AGF at *P* < 0.05. The value of each bar represents the mean ± SD of *n*=3.
